# Regulatory Role of PlaR (YiaJ) for Plant Utilization in *Escherichia coli* K-12

**DOI:** 10.1038/s41598-019-56886-x

**Published:** 2019-12-31

**Authors:** Tomohiro Shimada, Yui Yokoyama, Takumi Anzai, Kaneyoshi Yamamoto, Akira Ishihama

**Affiliations:** 1 Meiji University, School of Agriculture, Kawasaki, Kanagawa 214-8571 Japan; 2 Hosei University, Research Institute of Micro-Nano Technology, Koganei, Tokyo 184-0003 Japan; 3 Hosei University, Department of Frontier Bioscience, Koganei, Tokyo 184-8584 Japan

**Keywords:** Bacterial transcription, Transcriptional regulatory elements

## Abstract

Outside a warm-blooded animal host, the enterobacterium *Escherichia coli* K-12 is also able to grow and survive in stressful nature. The major organic substance in nature is plant, but the genetic system of *E. coli* how to utilize plant-derived materials as nutrients is poorly understood. Here we describe the set of regulatory targets for uncharacterized IclR-family transcription factor YiaJ on the *E. coli* genome, using gSELEX screening system. Among a total of 18 high-affinity binding targets of YiaJ, the major regulatory target was identified to be the *yiaLMNOPQRS* operon for utilization of ascorbate from fruits and galacturonate from plant pectin. The targets of YiaJ also include the genes involved in the utilization for other plant-derived materials as nutrients such as fructose, sorbitol, glycerol and fructoselysine. Detailed *in vitro* and *in vivo* analyses suggest that L-ascorbate and α-D-galacturonate are the effector ligands for regulation of YiaJ function. These findings altogether indicate that YiaJ plays a major regulatory role in expression of a set of the genes for the utilization of plant-derived materials as nutrients for survival. PlaR was also suggested to play protecting roles of *E. coli* under stressful environments in nature, including the formation of biofilm. We then propose renaming YiaJ to PlaR (regulator of plant utilization). The natural hosts of enterobacterium *Escherichia coli* are warm-blooded animals, but even outside hosts, *E. coli* can survive even under stressful environments. On earth, the most common organic materials to be used as nutrients by *E. coli* are plant-derived components, but up to the present time, the genetic system of *E. coli* for plant utilization is poorly understand. In the course of gSELEX screening of the regulatory targets for hitherto uncharacterized TFs, we identified in this study the involvement of the IclR-family YiaJ in the regulation of about 20 genes or operons, of which the majority are related to the catabolism of plant-derived materials such as ascorbate, galacturonate, sorbitol, fructose and fructoselysine. Therefore, we propose to rename YiaJ to PlaR (regulator of plant utilization).

## Introduction

Bacteria constantly monitor environmental conditions, and respond for adaptation and survival by modulating the expression pattern of their genomes. Transcription, the major regulation step in gene expression, is carried out by a single species of RNA polymerase (RNAP). The intracellular concentration of RNAP core enzyme in growing *Escherichia coli* K-12 W3110 strain is about 2,000 molecules per genome, which is less than the total of about 4,500 genes on its genome^1,2^. The expression pattern of a total of about 4,500 genes in its genome, however, can be modulated through alteration of the promoter selectivity of RNAP after interaction with two groups of the regulatory proteins, *i.e*., seven species of the promoter recognition subunit sigma^1,3,4^ and about 300 species of the DNA-binding transcription factors (TFs)^5,6^. Based on the protein structure of DNA-binding motifs, these TFs were classified into 54 families (5; TEC database [www.shigen.nig.ac.jp/ecoli/tec/]).

Up to the present time, more than 80% of the estimated 300 TFs in *E. coli* K-12 have been linked to at least one regulatory target gene or operon in its genome. The search for regulatory targets of these TFs has been carried out *in vivo* using both the ordinary molecular genetic approaches and the modern methodologies such as transcriptome using DNA microarrays and chromatin immunoprecipitation (ChIP) approaches. Using only *in vivo* analyses, however, it is difficult to get the complete set of regulatory targets because the binding *in vivo* of test TFs to their DNA targets is interfered with by both approximately 300 species of co-existing TFs and a number of nucleoid-associated DNA-binding proteins^5^. The regulatory targets of TFs identified *in vivo* often include indirectly regulated genes^1,7^, because the regulatory targets of one test TF often include the genes encoding other TFs, thereby forming a TF network hierarchy^5^. In order to identify the whole set of direct targets for each of these TFs, we have established the *in vitro* “Genomic SELEX (systematic evolution of ligands by exponential enrichment)” (gSELEX) screening system^8,9^. Since *E. coli* TFs generally bind to the recognition sequences located near the promoters of regulatory target genes and operons, gSELEX is one short-cut approach for the identification of regulatory targets under the direct control of a test TF^5^.

With the use of this systematic gSELEX screening system, we have succeeded in identifying the whole set of regulatory targets for more than 20 of hitherto uncharacterized TFs, designated as Y-TFs (for the list see Ishihama *et al*.^5^). Here we describe the whole set of regulatory targets for YiaJ, a poorly characterized IclR-family TF. YiaJ has been recognized as a repressor of the adjacently located *yiaKLMNOPQRS* gene cluster^10^. The only known function of this gene cluster is its participation in the utilization of an as yet unidentified carbohydrate that generates the intermediate L-xylulose^11^. The *yiaP* (*lyxK*) gene encodes a specific L-xylulose kinase for phosphorylation of 3-keto-L-gulonate that is derived from aspartic acid. The product, D-xylulose 5-phosphate, is considered to be degraded by a combination of three enzymes, SgbH (3-keto-L-gulonate-6-phosphate decarboxylase), SgbU (L-xylulose 5-phosphate 3-epimerase) and SgvE (L-ribulose-5-phosphate 4-epimerase) encoded by three downstream genes *yiaQ, yiaR* and *yiaS*^10,12^. The *yiaMNO* genes located in the middle of this operon have also been proposed as encoding the periplasmic transporter for uptake of unidentified osmoprotectants^13^ or the rare pentose L-xylulose (L-threo-2-pentulose or 2,3-dioxo-L-gulonate)^14^. Based on these observations, we predicted the involvement of the *yiaKLMNOPQRS* operon in the catabolism of ascorbate, a major product of plant fruits, and the participation of YiaJ in regulation of this operon. Beside the regulation of this *yiaKLMNOPQRS* operon for ascorbate utilization, YiaJ was also found to regulate a set of genes involved in utilization of other plant-derived materials such as galacturonate from plant pectin, sorbitol (sugar alcohol) in many edible fruits, and fructose and its Maillard reaction product (fructoselysine) in many vegetables. In addition, a number of genes for survival of *E. coli* K-12 under stressful conditions in nature are also found to be under the control of YiaJ. We then propose to rename YiaJ to PlaR (regulator of plant utilization).

## Results

### Search for PlaR-binding locations by gSELEX screening

In order to identify the regulatory targets of PlaR, we employed the gSELEX screening system^8,9^ using purified His-tagged PlaR and a mixture of 200–300 bp-long genome fragments from *E. coli* K-12 W3110 as the DNA substrate. PlaR-bound DNA segments were affinity-isolated using Ni-NTA agarose. This gSELEX screening was repeated up to six cycles. The original mixture of genome DNA fragments formed smeared bands on PAGE, but after repeated gSELEX screening, the PlaR-bound DNA formed sharper bands on PAGE, indicating the enrichment of specific DNA fragments with PlaR-binding activity.

**Figure 1:**
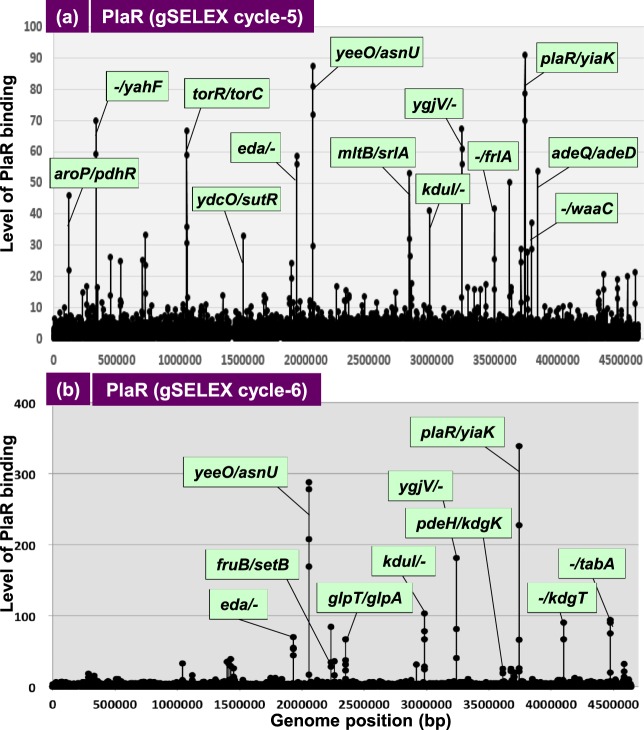
Mapping of PlaR-binding sites along the *E. coli* K-12 genome. (**a**) gSELEX screening for 5-cycles; (**b**) gSELEX screening for 6-cycles. gSELEX screening of PlaR-binding sequences was performed after which, a collection of DNA fragments was subjected to chip analysis using the tilling array of the *E. coli* K-12 genome. The y-axis represents the relative number of PlaR-bound DNA fragments, and the x-axis represents the position on the *E. coli* genome. The locations of PlaR-binding sites on the intergenic region are shown. The detailed PlaR-binding sites are listed in Fig. 2.

**Figure 2:**
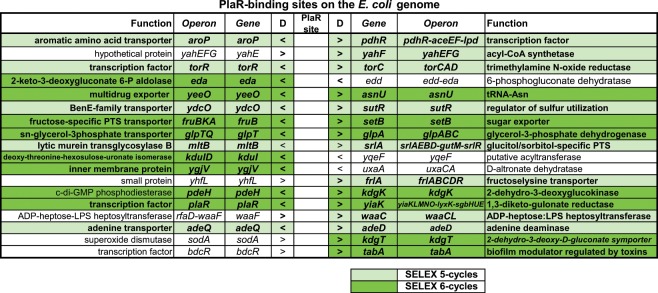
PlaR-binding sites on the *E. coli* genome were identified by using gSELEX-chip (see Fig. 1 for the gSELEX pattern). Possible regulation targets of PlaR are shown in bold, of which identified SELEX 5-cycles are shown under pale and dark green background, and SELEX 6-cycles are shown under dark green background.

To identify the PlaR-binding sites, affinity-isolated DNA segments were subjected to SELEX-chip (tilling array chip) analysis. After 5 cycles of gSELEX, more than 25 high-level peaks were identified (Fig. 1a), but after 6 cycles, the DNA fragments with weak affinity to PlaR were eliminated, resulting in about nine major peaks (Fig. 1b), which were all identified in the SELEX pattern after the 5th gSELEX cycle (for details see Fig. 2). This finding supports the prediction that DNA segments with higher affinity to test TF is enriched after repetition of gSELEX^8^. A total of 18 PlaR-binding sites were identified inside spacer regions after SELEX 5-cycles (Figs. 1a and 2, shown under pale and dark green) but only 10 high-affinity sites were identified after SELEX 6-cycles (Figs. 1b and 2, shown under dark green).

The high-level peak of PlaR binding was identified within two spacer regions of bidirectional transcription units, one between *yeeO* and *asnU* and the other between *plaR* and *yiaK* (Fig. 1b). The *yeeO* gene encodes a MATE (multi-antimicrobiotic drug and toxic compound extrusion)-family transporter while the *asnU* gene codes for tRNA-Asn. We then propose that the *plaR* gene product regulates the adjacent *yiaKLMNOPQRS* operon, which was once thought to be involved in the catabolism of L-lyxose^10^, a rare aldopentose present in bacterial glycolipids.

### Prediction of the regulatory targets of PlaR

Based on the pattern after 5-cycles of gSELEX, a total of 18 PlaR-binding sites were identified in intergenic spacer regions, of which 10 were inside bidirectional transcription units and 8 were located prior to one target gene but after another target gene (Fig. 2). Hence we concluded that there are between 18 and 28 regulatory targets of PlaR. The maximum of 28 targets can be classified into specific group of operons (for details see Fig. 2): 6 operons encoding specific TFs, PdhR, TorR, SutR, SrlR, FrlR and PlaR itself; 9 operons coding for transporters AroP, YdcO, YeeO, FruBKA, GlpT, SrlA, FrlA, AdeQ and KdgT; 10 operons coding for enzymes of carbon metabolism (*aceEF-lpd*, *yahFG*, *eda*, *glpABC*, *srlAEBD-gutM*, *kduI*, *frlABCD*, *kdgK, yiaKLMNO-lyxK-sgbHUE* and *adeD*); 3 genes for envelope formation (*mltB*, *ygjV* and *waaC*) and 2 target operons for response to external stresses (*torCAD* and *tabA*). The physiological roles of these genes or operons are described below (see DISCUSSION). Since as many as 6 TFs were predicted to be under the direct control of PlaR, a number of genes might be regulated indirectly by PlaR through these 6 TFs.

After 6-cycles of gSELEX, high-affinity PlaR-binding was focused onto ten sites (Figs. 1b and 2, shown under dark green background). The *yiaKLMNOPQRS* operon with the highest activity of PlaR binding was predicted to be involved in the catabolism of hitherto unidentified rare carbon sources such as L-lyxose^10^, L-xylulose^14^ and/or ascorbate in the case of *Klebisiella pneumonia*^15^. Some of the target genes are also involved in catabolism of rare carbon sources such as *eda* (or *kdgA*)*, kduID, kdgK*, and *kdgT* for D-galacturonate, a degradation product of pectin^16,17^, *fruBKA* for D-fructose^18^, *srlAEBD-gutM* for sorbitol^19^, *glpABC* for glycerol^20^ and *frlABCD* for fructocelysine^21^.

### Confirmation of PlaR-binding to the estimated target sites

**Figure 3:**
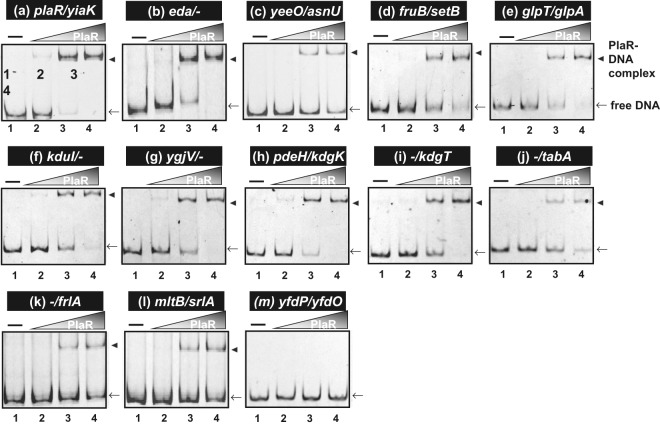
Confirmation of PlaR-binding *in vitro* to the regulatory targets: Gel shift assay. Purified PlaR was mixed with 0.5 pM of each DNA probe corresponding to the PlaR-binding regions shown in Fig. 2. PlaR (μM) was added: lane 1, 0; lane 2, 1; lane 3, 2.5; lane 4, 5. Filled triangles indicate the PlaR-DNA probe complex, and arrows indicates free probe.

To confirm the binding *in vitro* of PlaR to the target sites predicted based by the gSELEX screening, we carried out a gel shift assay for detection of PlaR-target DNA complexes. Fluorescent DNA probes were prepared for ten targets with high-affinity to PlaR binding (Fig. 3a–j) and other two other low-affinity targets, *mltB-srlA* and *frlA* (Fig. 3k,l). They were mixed with increasing concentrations of purified PlaR, and then the probe-PlaR mixtures were directly subjected to PAGE. All these twelve DNA probes formed PlaR concentration-dependent PlaR-DNA complexes, but the binding was not observed with a non-specific *yfdP-yfdO* intergenic probe used as an internal reference (Fig. 3m). Judging based on the amount of probes left unbound (located at the free DNA positions), the affinity of PlaR was the strongest for the *plaR-yiaK* spacer probe (Fig. 3a).

### Recognition sequence of PlaR

**Figure 4:**
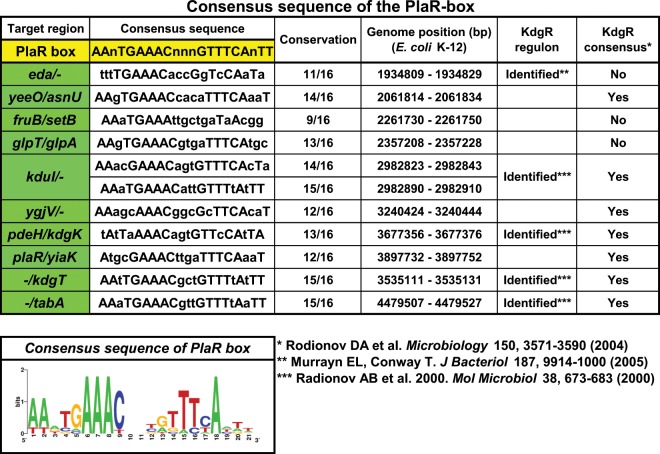
Consensus sequence of PlaR-box. Sequences of the probes with high level of PlaR-binding activity were analyzed using DMINDA 2.0 program (http://bmbl.sdstate.edu/DMINDA2/) and WEBLOGO (http://weblogo.berkeley.edu/logo.cgi) was used for matrix construction.

Based on the gSELEX patterns and the gel shift assays, 10 high-affinity regulatory targets were selected to search for the PlaR-binding sequence. After screening for a sequence conserved among all these PlaR targets, we identified one 21 bp-long palindromic sequence, ATnTGAAACnnnGTTCAnTT, for all of these targets (Fig. 4). This PlaR-box sequence within the spacer between the *yiaKLMNOPQRS* operon and the *plaR* gene overlapped with the experimentally identified promoter of the *yiaK* gene^10^. Based on the location of PlaR binding, we predicted that the *yiaK* operon is repressed by PlaR. In the case of the plant pathogenic bacterium *Erwinia chrysanthemi*, the IclR-family KdgR recognizes a sequence similar to the *E. coli* PlaR-box sequence and regulates a set of genes for pectin degradation^22^. *E. coli* K-12 is unable to utilize pectin, but once it is degraded by pectinases associated with as yet unidentified plant pathogens, *E. coli* K-12 is able to catabolize its product, D-galacturonate, using a set of genes, including KduI (YqeE; 5-dehydro-4-deoxyuronate isomerase), KduD (YqeD; 2-keto-3-deoxy-D-gluconate dehydrogenase) and Eda (KdgA; KHG/KDPG aldolase)^16^. Here these genes were identified as the regulatory targets of PlaR by gSELEX (see Figs. 1 and 2). Since these genes also contain the KdgR-box sequence (Fig. 4)^17,23,24^, we then propose co-regulation of these genes by both PlaR and KdgR. This possibility was examined as described below.

### Transcription regulation *in vivo* of predicted target genes by PlaR

**Figure 5:**
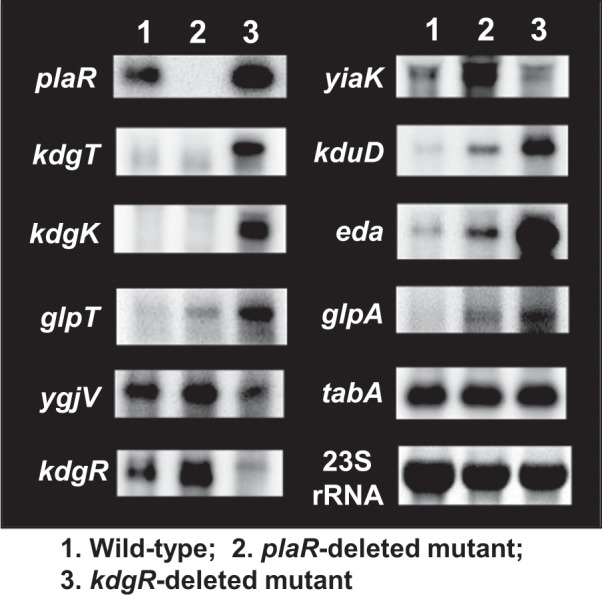
Influence of PlaR on transcription of the regulatory target genes. *E. coli* wild-type BW25113 (lane 1) and its *plaR*-defective mutant (lane 2) and *kdgR*-defective mutant (lane 3) were grown in M9-casamino acids (0.5%) medium at 37 °C with shaking under aerobic conditions. In the middle of exponential phase, total RNA was extracted from each culture and subjected to Northern blot analysis. DIG-labelled hybridization probes are shown on the left side of each panel. The amounts of total RNA analyzed were determined by measuring the intensity of ribosomal RNAs.

To examine the regulatory role of PlaR on transcription *in vivo* of the predicted targets, we next performed Northern blot analysis. Total RNA was prepared from exponential-phase cells of wild-type *E. coli* K-12 and its *plaR*-deleted mutant, and the level of mRNA was measured for ten representative target genes using DIG-labelled probes. In the absence of PlaR, marked increase in mRNA was observed for the 5′-proximal *yiaK* gene of the *yiaKLMNOPQRS* operon, the *kduD* gene of *kduID* operon encoding the genes for D-galacturonate catabolism of D-galacturonate (a degradation product of plant pectin), and the *eda* gene encoding Enter-Doudoroff aldolase, that is involved in the final step of D-galacturonate catabolism (Fig. 5, compare lanes 1 and 2). Significant increase was also observed for *glpA* coding for glycerol-3-phosphate dehydrogenase, and *glpT* encoding glycerol 3-phosphate:phosphate antiporter. The level of *ygjV* (inner membrane protein) mRNA in wild-type *E. coli* K-12 was higher than that in the *plaR*-defective mutant, and it further increased in PlaR-expressing strain. The expression level of *tabA* (toxin-antitoxin biofilm-inducing protein), however, apparently stayed constant, implying involvement of another as yet unidentified TF in the expression of the *tabA* gene. These observations altogether indicate that PlaR plays a repression role for these genes.

**Figure 6:**
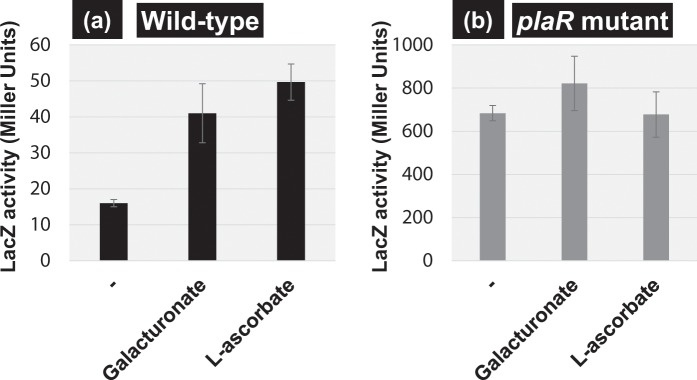
Repoter assay of the *yiaK* promoter. Reporter assay of the *yiaK* promoter was carried out using the *lacZ* reporter encoding *β*-galactosidase. Single copy *lacZ* gene reporter strains containing *yiaK-lacZ* was constructed both in wild-type strain (**a**) and *plaR* deleted strain (**b**). The strain was grown in M9 medium supplemented with 0.5% casamino acids. *β*-galactosidase activity was measured 1 h after addition of each effector in the middle of the exponential phase. 0.2% of α-D-galacturonate or 30 mM of L-ascorbate was supplemented, respectively. The data represent mean values from three separate experiments.

The regulatory role of PlaR on the *yiaK* promoter was also examined by using a LacZ single-copy reporter assay system. The expression of β-galactosidase was low in wild-type *E. coli* K-12 grown in M9-casamino acids medium (Fig. 6a, left bar). The LacZ activity, however, increased markedly in the mutant lacking the *plaR* gene (Fig. 6b), supporting the repressor function of PlaR for expression of the *yiaK* operon.

Since co-regulation was suggested between PlaR and KdgR, we also examined transcription level of the PlaR-target genes in the absence of KdgR by using Northern blot analysis. In the absence of KdgR, transcription increased for the *eda, kdgT, kdgK and kduD* genes, which were the known targets of KdgR. In addition, the increase of mRNA was also observed for the *glpT* and *glpA* gene (Fig. 5, compare lanes 1 and 3), implying the novel targets of KdgR. It is noteworthy that the level of *plaR* mRNA significantly increased in the absence of KdgR whereas *kdgR* mRNA increased in the absence of PlaR. This finding suggests a regulatory interplay between the two transcription factors, PlaR and KdgR.

### Role of PlaR on utilization of plant-derived nutrients

**Figure 7:**
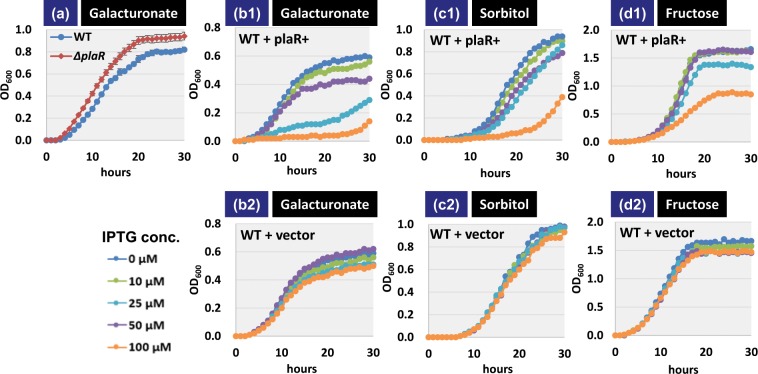
Influence of PlaR on cell growth in the presence of difference carbon sources. *E. coli* K-12 wild-type BW25113, *plaR* deleted mutant (**a**), and wild-type with IPTG-inducible PlaR-expression plasmid (b1 to d1) or vector plasmid pCA24N (b2 to d2) were grown in M9 medium containing 0.2% of galacturonate (**b**), sorbitol (**c**) or fructose (**d**). Various IPTG concentrations were added to evaluate the influence of PlaR expression level (b1 to d2). Cell growth was monitored by following the culture turbidity at 600 nm. The average of triplicate experiments is shown.

Most of the genes predicted to be under the control of PlaR were found not to be expressed under laboratory culture conditions. As an attempt to identify an environmental condition(s) or factor(s) that require PlaR and/or its regulatory target(s) for *E. coli* K-12 growth, we measured the rate of cell growth for wild-type *E. coli* K-12 BW25113 and its *plaR*-deletion mutant JW3546 in minimal M9 medium containing 0.2% of galacturonate, a breakdown product of pectin. The rate of cell growth was higher for the *plaR* mutant than the wild-type strain (Fig. 7a). This finding suggests that *E. coli* K-12 can utilize galacturonate as its sole carbon source, and in the complete absence of PlaR repressor, the cell growth further increased.

Excess expression of PlaR should repress the genetic system for utilization of galacturonate. To investigate this possibility, we over-expressed PlaR *in trans* by using the IPTG-inducible expression system. In fact, the IPTG dose-dependent expression of PlaR interfered markedly with the cell growth (Fig. 7b1), but this inhibition of cell growth was not observed in the presence of an empty vector (Fig. 7b2). Furthermore, as an attempt to detect the effect of PlaR on the utilization of other plant-derived nutrients, growth of PlaR-expressing strain was monitored in minimal M9 medium containing 0.2% each of sorbitol or fructose as a sole carbon source. The results indicated that over-expression of PlaR interfered with the cell growth (Fig. 7c1,d1), but not by the empty vector (Fig. 7d1,d2). These results altogether suggest that PlaR is involved in utilization control of plant-derived nutrients.

### Search for effectors controlling PlaR activity

**Figure 8:**
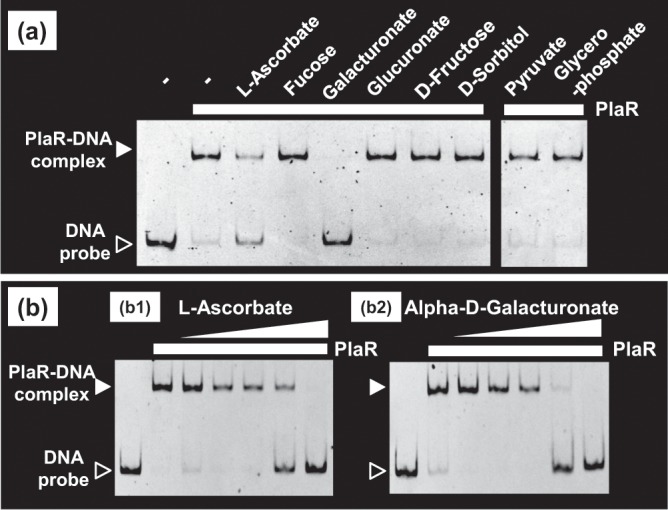
Search for inducer candidates for derepression of the *yiaK* promoter by PlaR. 2.5 uM of Purified PlaR was mixed with 0.5 pM of a DNA probe corresponding to the promoter sequence of *yiaK* gene. The *yiaK* promoter formed stable complexes with PlaR as shown in Fig. 3. Possible inducers affecting the *yiaK* promoter-PlaR complex formation, a variety of carbon sources were examined using the gel shift assay system. (Panel a) Eight species of carbon metabolite were tested at 10 mM concentrations. (Panel b) influence of two effective metabolites, ascorbate and D-galacturonate, was examined in details by increasing concentrations. (**b**) Ascorbate or D-galacturonate (mM) was added: lane 3, 1; lane 4, 2.5; lane 5, 5; lane 6, 10; lane 7, 25, respectively. Filled arrow indicates PlaR-*yiaK* promoter probe complex and open arrow indicates free probe.

PlaR was shown to repress some of the operons or genes for utilization of plant-derived nutrients. In order to identify a possible inducer ligand(s) for derepression of the *yiaK* operon (the major regulatory target of PlaR), we first tried to identify metabolites affecting the binding *in vitro* of PlaR to the *yiaK* promoter. First we tested 10 mM concentrations of various carbon sources, and found significant reduction of PlaR binding for two metabolites, L-ascorbate and α-D-galacturonate (Fig. 8a). However, D-glucuronate, another degradation product of pectin, did not affect PlaR binding to the *yiaK* promoter.

We then examined the possible influence of the two effective metabolites, L-ascorbate and α-D-galacturonate, on the induction of the *yiaK* operon. Both the metabolites induced a dose-dependent dissociation *in vitro* of PlaR from the PlaR-*yiaK* promoter complex (Fig. 8b). This finding indicates that both L-ascorbate and α-D-galacturonate play a regulatory role in controlling the activity of the PlaR repressor. To confirm the activation *in vivo* of PlaR by these two compounds, we carried out a *yiaK* reporter assay in wild-type and a *plaR*-deletion mutant. To identify the specificity of the effector ligand of PlaR inactivation, we measured the activity of the *yiaK* promoter in the presence of 0.2% of α-D-galacturonate or 30 mM of L-ascorbate (Fig. 6). In wild-type strain, the *yiaK* promoter was activated in the presence of α-D-galacturonate or L-ascorbate (Fig. 6a). In contrast, in *plaR* knock-out mutant, little activation of the *yiaK* promoter was observed (Fig. 6b). The effect of L-ascorbate found in this study is in good agreement with the reported modest activation of the *yiaK* promoter by L-ascorbate^10^. We then conclude that PlaR recognizes α-D-galacturonate and L-ascorbate as its effectors.

## Discussion

### gSELEX search for complete set of the regulatory targets of PlaR

Identification of the connections between TFs and their direct regulatory targets is a major bottleneck for modeling the transcriptional regulatory networks. The first step in such modeling is to make a complete list of regulatory targets of test TFs. After the identification of the whole set of genes on the genome, the transcription profile of entire genome became the front-line research for the model prokaryote *E. coli*. For this purpose, the transcription profile of the entire genome has been analyzed *in vivo* by using modern biotechnologies such as microarray-based transcriptome and antibody-based ChIP-chip analyses. Such *in vivo* approaches, however, include a number of unavoidable problems. For instance, all 300 TFs are not always present in *E. coli*^25^; some TFs are not always functional because of a lack of effector ligands; and TFs compete with each other and with other DNA-binding proteins in binding to targets on the genome^1,5,7^. Although genome regulation models have been constructed based upon experimental data obtained using varieties of *E. coli* strains^8^, but the number of TF genes common to all *E. coli* strains represents only a small fraction of the entire *E. coli* gene pool^26,27^.

To avoid the problems associated with *in vivo* approaches, we developed two-lines of *in vitro* approach: gSELEX (Genomic SELEX)^8,9^ and PS(promoter-specific)-TF screening systems^28,29^. One unique point of our *in vitro* screening systems is that all the materials used were all prepared from a single and the same *E. coli* K-12 W3110 type-A strain^30^. Here we employed this gSELEX system to screen the regulatory targets of an as yet uncharacterized YiaJ (renamed to PlaR). A total of minimum 18 and maximum 28 target genes were identified. The predicted target genes or operons were classified into several groups based on their biological functions.

### Utilization of plant-derived nutrients

*E. coli* is able to consume a variety of natural compounds as carbon sources through integration into its dedicated metabolic pathway. Nutrients that are more efficiently or quickly catabolized are utilized preferentially. The preference of nutrient utilization is determined by the molecular mechanisms such as catabolite repression, thereby forming the hierarchy^31,32^. Outside the natural host animals, plants are the most abundant source of nutrients for enterobacteria, but *E. coli* lacks some of the genetic systems for utilization of plant-derived nutrients. Otherwise such genetic systems are not expressed in *E. coll* under natural conditions.

**Figure 9:**
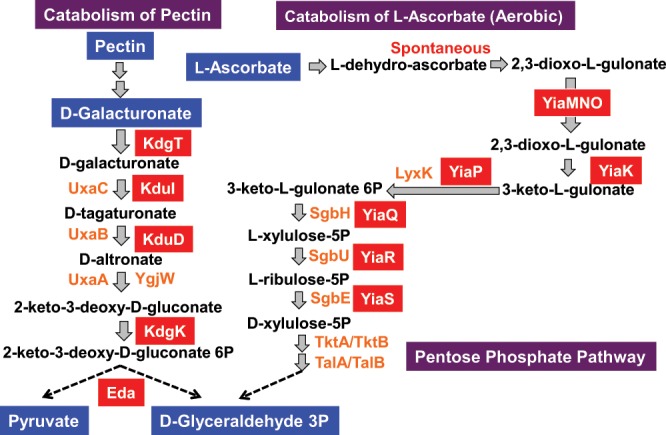
Participation of PlaR-target genes in the catabolism of D-galacturonate and L-ascorbate. The regulatory target genes were identified by gSELEX screening. These PlaR-target genes are involved in the catabolism of L-galacturonate and L-ascorbate. L-Galacturonate is derived from plant cell-wall pectin after digestion by pectinases from plant pathogens and transported into *E. coli* while L-ascorbate from fruits is spontaneously oxidized and taken up by *E. coli* through the YiaMNO importer. The genes under the direct control of PlaR are shown with under red background.

PlaR was identified as a repressor for the adjacently located *yiaKLMNOPQRS* gene cluster^10^. As noted above, this operon encodes a set of enzymes, which are involved in the catabolism of L-ascorbate and D-galacturonate (a product of plant pectin) (see Fig. 8). One major integral component of plant cell wall is pectin, a heterogeneous polysaccharide that is composed primarily of galacturonic acid, forming glycan matrix fibers. Pectin is degraded by a battery of pectinases for utilization as carbon source nutrients^33^. Resulting oligogalacturonide chains are transported into the periplasm space through anion-specific oligosaccharide KdgT porins^34^. The oligogalacturonides were further degraded into oligogalacturonides by the downstream pectinases, transported into the cytoplasm and ultimately degraded into pyruvate and 3-phosphoglyceraldehyde by the enzyme encoded by the *eda* gene (Fig. 9), which is also under the control of PlaR.

**Figure 10:**
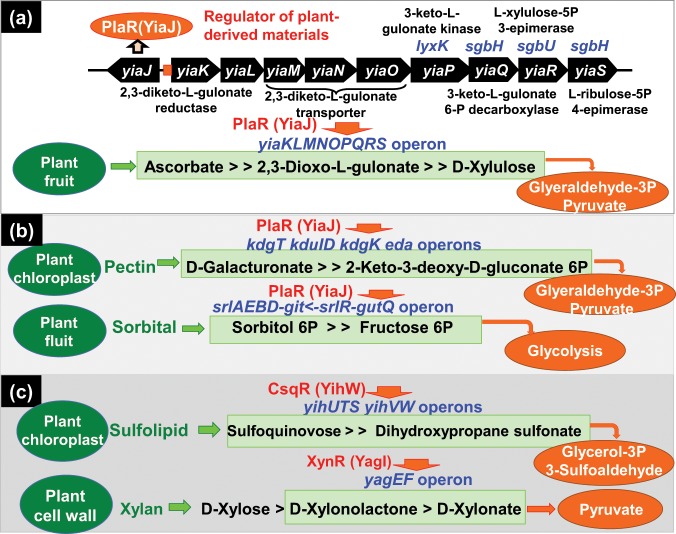
Regulatory roles of hitherto uncharacterized Y-TFs in utilization of plant-derived nutrients. After gSELEX screening of regulatory targets of hitherto uncharacterized TFs, we have identified the involvement of some TFs in regulation of a group of genes that participate in utilization of plant-derived materials. Here we identified the regulation by PlaR (renamed YiaJ) of genes for utilization of L-ascorbate from plant fruits (**a**). PlaR was also indicated to regulate genes participating in the utilization of D-galacturonate derived from plant cell-wall lectin and sorbital from fruit (**b**). Previously we identified the involvement of CsqR (renamed YihW) in the catabolism of fulfolipid from plant chloroplast (Shimada *et al*.^36^) and XynR (renamed YagI) in the catabolism of xylan from plant cell wall (Shimada *et al*.^35^) (**c**).

PlaR also regulates the *srlAEBDMR* operon, which is involved in transport and degradation of sorbitol that is abundant in fruits (Fig. 10). After gSELEX screening, we found that a number of Y-TFs participated in the regulation of genes required for utilization of plant-derived materials, including XynR (renamed YagI) for utilization of cell-wall xylan-derived D-xylose^35^, and CsqR (renamed YihW) for utilization of plant chloroplast-associated sulfolipid^36^ (Fig. 10). However, these genetic systems for utilization of such poor nutrients are not needed when there are sufficient high-quality nutrients as found under the ordinary laboratory culture conditions.

### Stress response and biofilm formation

Under nutrient-starved stressful conditions in nature, *E. coli* K-12 should express varieties of the protection system for survival. Plants provide with an excellent ecosystem for microorganisms. One survival strategy for bacteria under harsh environments is to attach to plants for colonization through adhesion to the cell surface or by forming biofilm inside plant tissues^37,38^. It is noteworthy that PlaR regulates a set of genes for response to stressful conditions in nature. TorSR TCS, one of the major regulatory targets of PlaR, is involved in the regulation of the *torCAD* operon for anaerobic respiration on TMAO (trimethylamine N-oxide)^39^. TorR is also involved in the activation of alkaline stress defense and represses the acid stress response^40^. AdeD, a substrate-specific adenine deaminase, is activated in the presence of either Mn^2+^ or Fe^2+^ ^41^. Ferrous iron-containing enzyme carries the catalase activity, which generates superoxide. YeeO, a multidrug and toxic compound extrusion (MATE) protein, plays a role in cellular homeostasis by secreting metabolic wastes, excess riboflavin, FMN and FAD^42^.

L-Ascorbic acid is the lactone of 2,3 dienol-L-gulonic acid, which is structurally similar to the hexose sugars, and thus L-ascorbate could serve as a nutrient for bacterial growth^43^. In nature, L-ascorbic acid is abundant in fruits, but when it is outside cells, its dienol group is easily oxidized, generating reducing powers, but the hydrogen peroxide produced causes bactericidal effects. Hence ascorbate serves to poise the oxidation-reduction potential for bacterial growth. In fact, *E. coli* K-12 is unable to grow under aerobic conditions if L-ascorbate is the sole carbon source (Shimada T, unpublished).

Nonlethal concentrations of ascorbate inhibit bacterial quorum sensing and biofilm development. Ascorbate enhances the killing effect of antibiotics presumably because it inhibits the formation of biofilm^44,45^, which offers the protection against antimicrobial agents^46^. Biofilm is covered with a hydrophobic EPS coating, which retards the penetration of antimicrobial agents and confers biofilm resistance^47^. Once the EPS content is reduced, bacterial cells become exposed to any external factors including antibiotics, and are more susceptible to their killing effects. PlaR is thought to regulate the *tabA* gene that modulates the level of biofilm formation. Toxin-dependent expression of the *tabA* gene inhibits biofilm dispersal through inhibition of biofilm dispersal^48^. H-NS silences a wide-range genes including the genes related to biofilm formation, and thus the deletion of *hns* decreases biofilm formation^49^. The silencing function of H-NS is interfered with a set of global regulators with anti-silencing role such as LeuO^50^. The function of H-NS is also controlled through interaction of small regulatory proteins, Cnu, Hha and YmoB^51^. The *cnu* gene was suggested to be under the direct control of PlaR (see Fig. 2). Likewise PlaR-regulated YisMNS transporter influences the adhesive properties of *E. coli*, leading to control of biofilm formation.

## Materials and Methods

### Bacterial strains

*E. coli* DH5α was used for plasmid amplification. *E. coli* BL21 (DE3) was used for PlaR expression and purification. *E. coli* K-12 W3110 type-A for genome DNA segments used for gSELEX. *E. coli* K-12 BW25113^52^ and its single-gene deletion mutants, JW3546 (*plaR*) and JW1816 (*kdgR*)^53^, were obtained from the *E. coli* Stock Center (National Bio-Resource Center) for the assays of cell growth, promoter activity and Northern blotting. JW3546 lacking a Km marker was constructed in this study. Cells were grown in LB or M9 minimal media with supplemented 0.2% of galacturonate or 0.5% of Casamino acids at 37 °C under aeration with constant shaking at 150 r.p.m. When necessary, 20 μg ml^−1^ kanamycin was added into the medium. Cell growth was monitored by measuring the turbidity at 600 nm.

### Purification of PlaR protein

Expression plasmid pPlaR used for over-expression and purification of His-tagged PlaR was constructed essentially according to the standard procedure^9^. For construction of plasmid for PlaR expression, a DNA fragment corresponding to the PlaR-coding sequence was amplified by PCR and cloned into pET21a(+) (Novagen) between NdeI and NotI sites, leading to construct pPlaR. For protein expression, pPlaR plasmid was transformed into *E. coli* BL21 (DE3). Transformants were grown in LB medium and PlaR expression was induced by adding 1 mM IPTG in the middle of exponential growth phase. PlaR protein was purified by the affinity purification procedure with use of a Ni-nitrilotriacetic acid (NTA) agarose column. The affinity-purified PlaR protein was stored frozen in the storage buffer at −80 °C until use. Protein purity was more than 95% as checked by SDS-PAGE.

### gSELEX screening of PlaR-binding sequences

The gSELEX screening was carried out according to the standard procedure^8,9^. The SELEX cycle was repeated five and six times, respectively, for enrichment of PlaR-binding sequences. DNA was isolated from DNA-PlaR complexes by PAGE and PCR amplified. Sequence analysis of PCR-amplified PlaR-bound DNA fragments was performed by gSELEX-chip (microarray chip) method. Mapping of SELEX fragments along the *E. coli* genome was also performed by SELEX-chip system by using a 43,450-feature DNA microarray (Oxford Gene Technology). The genomic SELEX sample obtained with use of PlaR was labeled with Cy3, while another SELEX sample obtained in the absence of PlaR addition was labeled with Cy5. After hybridization of samples to the DNA tiling array, the Cy3/Cy5 ratio was measured and the peaks of scanned patterns were plotted against the positions of DNA probes along the *E. coli* K-12 genome.

### Gel shift assay

The gel shift assay was performed as described previously^54^. The probes of promoter region were synthesized using a set of primers (Table S1a), BW25113 genome as a template, and Ex *Taq* DNA polymerase (TAKARA). For gel shift assays, 0.5 pmol each of the probes was incubated at 37 °C for 20 min with various amounts of PlaR in 15 ml of gel shift buffer consisting of 10 mM Tris-HCl, pH 7.8 at 4 °C, 150 mM NaCl, and 3 mM Mg acetate. In the case of addition of chemical compounds, 1 ml of various concentrations of chemical compounds was added and incubated for another 20 min. After addition of a DNA dye solution, the mixture was directly subjected to 5% PAGE. The probe DNA in gels was stained by Gel-Red (Biotium) and detected using LAS-4000 IR multi-colour (GE healthcare).

### Consensus sequence analysis

To analyzed the PlaR binding sequence, a set of PlaR binding sequence identified by gSELEX-chip was analyzed by using DMINDA 2.0 program^55^. Sequences were aligned and consensus sequence logo was created by weblogo (http://weblogo.berkeley.edu/logo.cgi).

### Northern blotting assay

Total RNAs were extracted from exponentially growing *E. coli* cells (OD_600_ = 0.3) in M9-casamino acids (0.5%) media by the hot phenol method. RNA purity was checked by electrophoresis on 1.5% agarose gel in the presence of formaldehyde followed by staining with Methylene blue. Northern blot analysis was performed essentially as described previously^54^. DIG-labeled probes were prepared by PCR amplification using BW25113 genomic DNA as template, DIG-11-dUTP (Roche) and dNTP as substrates, gene-specific forward and reverse primers (Table S1b), and Ex Taq DNA polymerase (Takara). Total RNAs (2 μg) were incubated in formaldehyde-MOPS (morpholinepropanesulfonic acid) gel-loading buffer for 10 min at 65 °C for denaturation, subjected to electrophoresis on formaldehyde-containing 2% agarose gel, and then transferred to nylon membrane (Roche). Hybridization was performed with DIG easy Hyb system (Roche) at 50 °C overnight with a DIG-labeled probe. For detection the DIG-labeled probe, the membrane was treated with anti-DIG-AP Fab fragments and CDP-Star (Roche), and the image was scanned with LAS-4000 IR multi-colour (GE healthcare).

### Reporter assay of *yiaK* promoter activity

*yiaK* promoter fragment approximately 400 bp in length between initiation codon and 400 bp upstream sequence were amplified by PCR using a pair of primers (Table S1c), and cloned into pRS551 plasmid vector^56^. The single-copy *lacZ* (β-galactosidase) gene reporter strains containing *yiaK-lacZ* were constructed using λRS45 phage vector, as described previously^57^. The recombinant phage-containing *yiaK* promoter-*lacZ* fusion was isolated from the resulting phage lysate, and used to infect *E.coli* BW25113, JW3546 lacking a Km marker for screening of kanamycin-resistant. Single-copy *yiaK* promoter-*lacZ* fusion strains were grown in M9-casamino acids (0.5%) media and β-galactosidase activity was measured from exponentially growing *E. coli* cells (OD_600_ = 0.3) using ONPG as a substrate, as described previously^57^.

## Supplementary information

Dataset 1.
