# Author Correction: Regulatory Role of PlaR (YiaJ) for Plant Utilization in *Escherichia coli* K-12

**DOI:** 10.1038/s41598-020-59905-4

**Published:** 2020-02-14

**Authors:** Tomohiro Shimada, Yui Yokoyama, Takumi Anzai, Kaneyoshi Yamamoto, Akira Ishihama

**Affiliations:** 1 Meiji University, School of Agriculture, Kawasaki, Kanagawa 214-8571 Japan; 2 Hosei University, Research Institute of Micro-Nano Technology, Koganei, Tokyo 184-0003 Japan; 3 Hosei University, Department of Frontier Bioscience, Koganei, Tokyo 184-8584 Japan

Correction to: *Scientific Reports* 10.1038/s41598-019-56886-x, published online 31 December 2019

The original version of this Article contained errors in the Abstract.

“Outside a warm-blooded animal host, the enterobacterium *Escherichia coli* K-12 is also able to grow and survive in stressful nature. The major organic substance in nature is plant, but the genetic system of *E. coli* how to utilize plant-derived materials as nutrients is poorly understood. Here we describe the set of regulatory targets for uncharacterized IclR-family transcription factor YiaJ on the *E. coli* genome, using gSELEX screening system. Among a total of 18 high-affinity binding targets of YiaJ, the major regulatory target was identified to be the *yiaLMNOPQRS* operon for utilization of ascorbate from fruits and galacturonate from plant pectin. The targets of YiaJ also include the genes involved in the utilization for other plant-derived materials as nutrients such as fructose, sorbitol, glycerol and fructoselysine. Detailed *in vitro* and *in vivo* analyses suggest that L-ascorbate and α-D-galacturonate are the effector ligands for regulation of YiaJ function. These findings altogether indicate that YiaJ plays a major regulatory role in expression of a set of the genes for the utilization of plant-derived materials as nutrients for survival. PlaR was also suggested to play protecting roles of *E. coli* under stressful environments in nature, including the formation of biofilm. We then propose renaming YiaJ to PlaR (regulator of plant utilization). The natural hosts of enterobacterium *Escherichia coli* are warm-blooded animals, but even outside hosts, *E. coli* can survive even under stressful environments. On earth, the most common organic materials to be used as nutrients by *E. coli* are plant-derived components, but up to the present time, the genetic system of *E. coli* for plant utilization is poorly understand. In the course of gSELEX screening of the regulatory targets for hitherto uncharacterized TFs, we identified in this study the involvement of the IclR-family YiaJ in the regulation of about 20 genes or operons, of which the majority are related to the catabolism of plant-derived materials such as ascorbate, galacturonate, sorbitol, fructose and fructoselysine. Therefore, we propose to rename YiaJ to PlaR (regulator of plant utilization).”

now reads:

“Outside a warm-blooded animal host, the enterobacterium *Escherichia coli* K-12 is also able to grow and survive in stressful nature. The major organic substance in nature is plant, but the genetic system of *E. coli* how to utilize plant-derived materials as nutrients is poorly understood. Here we describe the set of regulatory targets for uncharacterized IclR-family transcription factor YiaJ on the *E. coli* genome, using gSELEX screening system. Among a total of 18 high-affinity binding targets of YiaJ, the major regulatory target was identified to be the *yiaLMNOPQRS* operon for utilization of ascorbate from fruits and galacturonate from plant pectin. The targets of YiaJ also include the genes involved in the utilization for other plant-derived materials as nutrients such as fructose, sorbitol, glycerol and fructoselysine. Detailed *in vitro* and *in vivo* analyses suggest that L-ascorbate and α-D-galacturonate are the effector ligands for regulation of YiaJ function. These findings altogether indicate that YiaJ plays a major regulatory role in expression of a set of the genes for the utilization of plant-derived materials as nutrients for survival. PlaR was also suggested to play protecting roles of *E. coli* under stressful environments in nature, including the formation of biofilm. We then propose renaming YiaJ to PlaR (regulator of plant utilization).”

