# Supplementary material for: Regulatory Role of PlaR (YiaJ) for Plant Utilization in Escherichia coli K-12
Source: Sci Rep. 2019 Dec 31;9:20415. doi: 10.1038/s41598-019-56886-x (PMC6958661; doi:10.1038/s41598-019-56886-x)
Supplement: Supplementary file 1 — Dataset 1. [file 41598_2019_56886_MOESM1_ESM.pdf]

Table S1. Primers used in this study

"Regulatory Role of PlaR (YiaJ) for Utilization of Plant-derived Nutrients in *Escherichia coli* K-12."

Tomohiro SHIMADA, Yui YOKOYAMA, Takumi ANZAI, Kaneyoshi YAMAMOTO and Akira ISHIHAMA

Table S1. Primers used in this study

a. Gel shift assay

**plaR/yiaK-F**	CAGTTCAATGGCGCGATCCATCAT
**plaR/yiaK-R**	TTTGTCGCGTCCTGATGGTAG
**eda-F**	AAGTGACTCTGCGTACCGAGTGT
**eda-R**	TTACAGCTTAGCGCCTTCTACAGC
**yeeO/asnU-F**	CCATAATGACCATATTGAAG
**yeeO/asnU-R**	CATCGTTGTTGAGGCACATCT
**fruB/setB-F**	GTCAGGTCAAACGATTTC
**fruB/setB-R**	CTGGAACATAGTTCTCCTCT
**glpT/glpA-F**	TAAAAATACTCAACATTGATAGC
**glpT/glpA-R**	ATCACGTCACTTGATTGC
**kduI-F**	TTCTCTGGTTCATGAAATG
**kduI-R**	ATGCTCTGTCTTACGTCC
**ygjV-F**	CTGAACTTGTTAAGTGCG
**ygjV-R**	GATAAATCCTTAGCAGGTATG
**pdeH/kdgK-F**	CTAACGCTAATTTTTTACAGATC
**pdeH/kdgK-R**	TTATCATCAAAGATGTCCTG
**kdgT-F**	TGCTTACTACCTGAAATTCC
**kdgT-R**	TTATCTGCATTTCCATCAC
**tabA-F**	GTTCTTGAAGGTATTCATAGTC
**tabA-R**	TGAAGATTATGAATATTTCCG
**frlA-F**	GTTGAGCTGGCGGCTACGCT
**frlA-R**	ACGCCGATTTCCCGACGCCCT
**mltB/srlA-F**	TTCGGGTTATTAGCGAAATC
**mltB/srlA-R**	GCACCATGAGTAATGGTTTC
**yfdO/yfdP-F**	CTCAAATCATCCTGCGTTC
**yfdO/yfdP-R**	TTTGCTGCTCCTCATCAT

b. Northern blotting

**plaR-F**	TTCGCTGAATATCATTCATATC
**plaR-R**	GTAAATCCCAGTTCATTAGAAATAG
**kdgR-F**	AAAACGTCGATTTAATTCGTAG
**kdgR-R**	AGAACGGATAGTCGTGATAAC
**yiaK-F**	TCGATATGTCGATGTCGATG
**yiaK-R**	TCATAACGCCTGGATTTTGG
**kdgT-F**	AGTACGGCACAAAAGAAGAAG
**kdgT-R**	CTGCTCTGGCTTTGACTTTAC
**kduD-F**	TGCGAAAGATTGATGGTATTC
**kduD-R**	GTATAACCATTCACATAATCTGAAG
**kdgK-F**	GAATTTCGATTATCTCTACCTGAG

**kdgK-R**	GATACTGAATAACGGTACTTGC
**eda-F**	GTGGTAAAAAAACTGGAACAC
**eda-R**	TTACAGCTTAGCGCCTTCTAC
**glpT-F**	TACTGCCGAACAAACTGCTG
**glpT-R**	TTCTTGCAGTAATTGTTCATG
**glpC-F**	TAGGTAAAGATTTAATTAAAGTGCTC
**glpC-R**	AATCTGCCATTTACAGGTTTC
**ygjV-F**	ATCGCCTTTCTGATTGGTATC
**ygjV-R**	CCAGAAACGAATGATATTCAG
**tabA-F**	ATCATCGGAAATATTCATAATC
**tabA-R**	AGCATCTTCACTACTGCTTTG

c. Promoter assay

**yiaK-F**	CCGGAATTCGTACTCTTATTTAAACCAGCC
**yiaK-R**	CGCGGATCCCCCAGGCTTCCTTGTTTTTT
